# In Vitro Antimicrobial Activity of *Piper retrofractum* Fruit Extracts against Microbial Pathogens Causing Infections in Human and Animals

**DOI:** 10.1155/2020/5638961

**Published:** 2020-02-14

**Authors:** Wattana Panphut, Tanakwan Budsabun, Pakkakul Sangsuriya

**Affiliations:** ^1^Industrial Microbiology Program, Faculty of Science and Technology, Suan Sunandha Rajabhat University, Bangkok, Thailand; ^2^National Center for Genetic Engineering and Biotechnology (BIOTEC), National Science and Technology Development Agency (NSTDA), Klong Luang, Pathumthani, Thailand

## Abstract

Long pepper (*Piper retrofractum* Vahl) is a Thai medicinal herb which has been used as one of the common ingredients in variety of Thai foods. Here, we investigated antimicrobial activities of crude bioactive metabolites extracted from fruits of *P. retrofractum* against 10 pathogenic organisms (bacteria and yeast) causing opportunistic infections in human or animals including *Bacillus subtilis* ATCC6633, *Staphylococcus aureus* ATCC25923, *Enterococcus faecalis* ATCC2921, *Escherichia coli* ATCC25922, *Klebsiella pneumonia* TISTR1843, *Pseudomonas aeruginosa* ATCC741, *Salmonella typhi* (clinical isolate), *Vibrio parahaemolyticus* (XN98 and 5HP), and *Candida albicans* ATCC90020. The results of disk diffusion test showed that the extract from methanol solvent exhibited greater antibacterial activity than other solvents with inhibition zones ranging from 0.5 to 8.0 mm, respectively. Subsequently, minimal inhibition concentration (MIC) determined by the colorimetric assay confirmed that methanol extracts showed consistent results with disk diffusion method. In summary, *in vitro* assays suggest that methanol is the best solvent for extraction of bioactive metabolites from *P. retrofractum* fruits. This crude extract can inhibit the majority of human and animal pathogens. This opens up a potential use of pepper fruits in prevention of food-contaminating microorganisms.

## 1. Introduction

Drug resistance of infectious microorganisms has been reported worldwide [1]. The most prevalent resistant species are the methicillin-resistant *Staphylococcus aureus* (MRSA), the extended-spectrum *β*-lactamase (ESBL)-producing *Escherichia coli*, *Klebsiella pneumoniae*, and multi-drug resistant *Pseudomonas aeruginosa* [2]. In addition, the consumption of food-contaminating microorganisms, such as *Enterococcus faecalis*, *Escherichia coli, Salmonella typhi*, and *Vibrio parahaemolyticus*, can pose a serious threat to human health. The existence of these microorganisms causes spoilage and often a food-borne disease [3]. Candidiasis is a fungal infection caused by *Candida albicans* that can affect areas such as skin, genitals, throat, mouth, and blood circulation system. It is caused by the overgrowth of *C. albicans* [4]. Candida infections can be difficult to treat and can reoccur after treatment. In people with weakened immune systems, candidiasis can be life threatening if it passes into the blood and spreads to vital organs [5]. Infection of *V. parahaemolyticus* causes acute hepatopancreatic necrosis disease (AHPND) in cultivated shrimp farm [6]. The disease spread rapidly and caused significant losses in Southeast Asian shrimp farms since 2009 [7].

Long pepper, or *Piper retrofractum* Vahl, is a flowering vine in the family Piperaceae, cultivated for its fruit. The dried fruit has long been used as a spice and seasoning. Thailand is located in tropical humidity zone which provides the best condition for growing *P. retrofractum*, especially in the central part of Kanchanaburi, Ratchaburi, Phetchaburi, and Chanthaburi provinces. Locals use *P. retrofractum* fruit as a key ingredient in various recipes of their traditional medicine and foods. There has been no report of toxicity from long pepper. Fruit of long pepper was analyzed for its chemical profile. Extracted bioactive metabolites from *P. retrofractum* were found to contain alkaloid component, amide derivative with antiflatulent, expectorant, antitussive, antifungal, and appetizing properties. In addition, it was reported to possess gastroprotective and cholesterol-lowering properties which could be useful for application in traditional medicine [8–10].

The aim of this research is to extract metabolites from long pepper using different solvents and to perform *in vitro* antimicrobial tests against various pathogenic bacteria and yeast.

## 2. Materials and Methods

### 2.1. Plant Extract Sample

**Figure 1:**
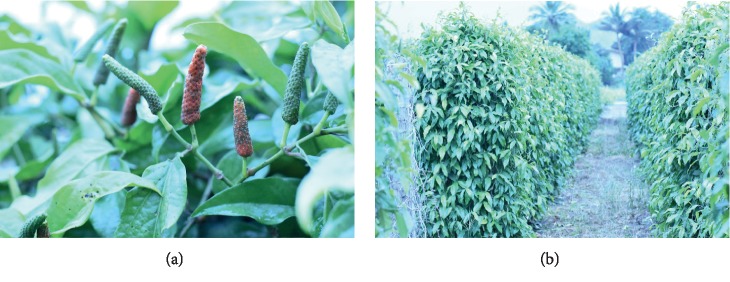
(a) Fruit of *P. retrofractum* Vahl consisting of many minuscule fruits each about the size of 5 cm with poppy seeds embedded in the surface of a flower spike; the ripe fruit turns to a red-orange color. (b) A garden of long pepper as a climbing plant and it is held together with a pillar.

Fresh fruits of *Piper retrofractum* Vahl (long pepper) were purchased from the botanical garden in Kanchanaburi, Thailand (Figure 1). The samples were ground in a blender. Each 250 g of the sample was then extracted with 500 mL of individual solvents including hexane, isopropanol, acetonitrile, dichloromethane, and methanol. The extraction was performed in a one-litter screw cap bottle and orbit-shaked at 150 rpm for 48 hrs at room temperature. Then, the samples were filtrated by passing through Whatman No. 1 and the residue was used for cascade extraction with respective organic solvent. The filtration of each solvent was evaporated by rotary evaporator (Rotavapor® R-300, BUCHI) to remove the solvent and dried in desiccator. The crudes were weighed and dissolved in DMSO (dimethyl sulfoxide) for the next experiments.

### 2.2. Microbial Cultures

The bacteria and fungi selected for this study are mostly human pathogens: Gram-positive bacteria, *Bacillus subtilis* ATCC6633, *Enterococcus faecalis* ATCC2921, and *Staphylococcus aureus* ATCC25923; Gram-negative bacteria, *Pseudomonas aeruginosa* ATCC741, *Klebsiella pneumonia* TISTR1843, *Escherichia coli* ATCC25922, *Salmonella typhi* (clinical isolate, Mahidol University, Thailand), *Vibrio parahaemolyticus* (5HP and XN89), and yeast *Candida albicans* ATCC90020. The culture collections obtained from Suan Sunandha Rajabhat University and two *V. parahaemolyticus* isolates were kindly provided by Center for Shrimp Molecular Biology and Biotechnology (Centex Shrimp), Mahidol University, Thailand.

### 2.3. Antimicrobial Susceptibility Testing by the Disk Diffusion Method

The crude extracts of *P. retrofractum* fruits were tested against the above pathogens by the disk agar diffusion method. The method for antibacterial disk diffusion susceptibility follows manual of antimicrobial susceptibility testing guidelines [11], and antifungal *C. albicans* disk diffusion susceptibility testing follows manual for antifungal disk diffusion susceptibility testing of yeasts in NCCLS guideline in 2004 [12]. All crude extracts were dissolved in dimethyl sulfoxide (DMSO). For initial screening, 40 *µ*l of the extract was loaded onto each Whatman No. 1 filter paper disk (Ø, 6 mm) and air-dried for 20 min. These bacteria were grown on Mueller–Hinton agar (MHA) medium (pH 7.3) except *Vibrio* spp. which were supplemented with 1.5% w/v NaCl and yeast using Sabouraud dextrose agar (SDA). Agar media were poured into the plates to uniform depth of 5 mm and allowed to solidify. The microbial suspensions were prepared by spectrophotometer using culture broth with adding sufficient sterile medium to adjust the transmittance to that produced by a 0.5 McFarland standard match to an optical density (OD) 0.1 at 625 nm wavelength. This procedure will yield bacterial stock suspension 1 × 10^8^ cfu/ml and yeast stock suspension of 1 × 10^6^ to 5 × 10^6^ cfu/ml. The microbial suspension was streaked over the surface of media using a sterile cotton swab to ensure the confluent growth of the organism. The disks used were Whatman® No. 1 papers, 6 mm in diameter, which were then aseptically applied to the surface of the agar plates at well-spaced intervals. The plates were incubated at 37°C for 24 h and observed growth inhibition zones, including the diameter of the disks, were measured. Control disks were impregnated with 10 *µ*l of the solvent DMSO.

### 2.4. Determination of Minimum Inhibitory Concentration (MIC)

The crude extract has shown significant antimicrobial activities in disk diffusion method which were selected for determination of MIC using the broth microdilution assay following NCCLS M07-A09 guideline [13] with minor modification. The microbial suspension was prepared by spectrophotometer OD 0.1 at 625 nm wavelength (0.5 McFarland standard) and contained approximately 1 × 10^8^ cfu/mL and yeast suspension of 1 × 10^6^ to 5 × 10^6^ cfu/mL as starting inoculums. The crude extract of serial two-fold dilutions with 100 *µ*l medium in 96-well plates was used, and then the inoculums were applied in each well with a final concentration 1 × 10^5^ cfu/ml for bacteria and 0.5–2.5 × 10^3^ cfu/mL for *C. albicans.* The microplate was incubated at 37°C for 24 hr (for bacteria) or 48 hr (for *C. albicans*). After incubation, 30 *µ*L tetrazolium salt (2,3,5-triphenyltetrazolium chloride, Merck), 0.02 *µ*g/mL, was added into each well. Colorimetric interpretation was performed after 1 hr incubation; the MIC was read as the lowest concentration of antimicrobial agent at which no color change occurred [14, 15].

## 3. Results

### 3.1. Medicinal Plant *Piper retrofractum* Vahl Morphology

**Figure 2:**
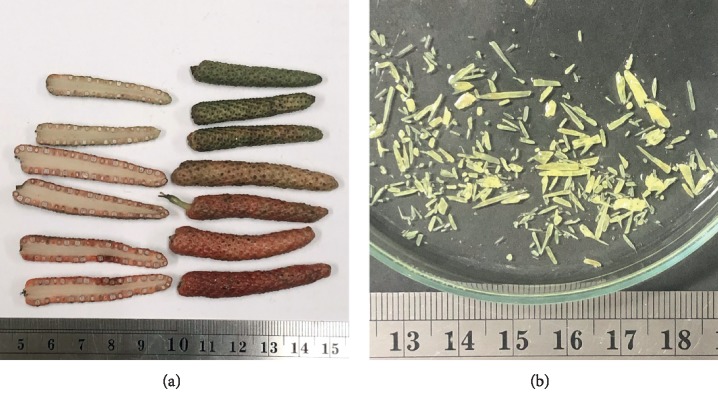
The multiple fruit of *P. retrofractum* Vahl when ripe changes to a red-orange color and consists of many minuscule seeds embedded in the same receptacle (a). The column chromatography shows purified pale yellow needle-like crystal 3–12 mm alkaloid from dichloromethane and isopropanol extraction of *P. retrofractum* Vahl (b).

*P. retrofractum* Vahl is a dicotyledon flower plant belonging to the Piperaceae family. It has a radical root when growing from the seed but in agriculture aerial stem transplantation is used which develops fibrous root from the pericycle layer. *P. retrofractum* had an adventitious root as climbing root at the node of aerial stem that was the spot-characteristic of climber herbaceous plant (Figure 1). Simple leaf is composed of lamina as lanceolate type with 10 × 5 cm area, and the leaf base is obtuse having the entire of margin. Leaf texture was glabrous indumentum, chartaceous, succulent phyllotaxy, and small rod petiole 5–7 cm long without stipule (Figure 1(a)). IT was a perfect flower, apetalous, tubular with androecium, and gynoecium syncarpous carpel axial placentation. Fruits of *P. retrofractum* Vahl are finger shaped with a length of 4.5–5.2 cm and a 0.3–0.5 cm diameter. It is a multiple fruit development and has multiple seed orientation surrounded on the same receptacle. The young fruit is of green color and a small size and the ripe fruits become of a red-orange color and consist of many minuscule seeds embedded in the same receptacle as shown in Figure 2(a). This is the main source of herbal material for solvent extraction for future steps.

### 3.2. Highest Yield of Crude Extract Found in Isopropanol Solvent

Using the same extraction protocol with equal amounts of solvents and fresh pepper fruits, five different solvents yielded dried crude production from 5.47 to 12.38% w/w (Table 1). Isopropanol solvent gave a highest yield of 12.38% (w/w), followed up by dichloromethane 11.15% (w/w) and acetonitrile 11.15% (w/w). Lower yields 5.475% (w/w) was produced in methanol solvents, respectively (Table 1).

The column chromatography was performed after crude extraction using silica gel 60 (0.015–0.040 mm, Merck). Dichloromethane extraction and isopropanol extraction were giving pale yellow needle-like crystal 3–12 mm and unique aroma of alkaloid, shown in Figure 2(b). The result of liquid chromatography mass spectrophotometry (LCMS, micrOTOF, Bruker) was analyzed and a pure crystal with 308.1297 g/mol molecular weight was produced, and this substance was shown to be a dimer substance form with 593.2979 g/mol molecular weight.

### 3.3. Solvent Extract Inhibited the Majorities of Tested Pathogens by Agar Disk Diffusion Test

Antimicrobial susceptibility testing results of the five different solvent extracts and a control antibiotic (ampicillin) against 10 pathogenic microorganisms were presented in Table 2. Among the five solvents used, methanol gave the best inhibition result. Methanol extract inhibited 9/10 of the tested pathogens with inhibition zone in the range from 0.5 to 8 mm. Inhibition zones for *C. albicans* ATCC90020, *S. aureus* ATCC25923, *P. aeruginosa* ATCC741, and *E. faecalis* ATCC2921 were 8.0, 6.0, 4.0, and 3.5 mm, respectively. No inhibition was observed in the *K. pneumonia* TISTR1843 isolate and it was very weak (0.5 mm) in *B. subtilis* ATCC6633 and *V. parahaemolyticus* 5HP. Remaining isolates (*E. coli* ATCC25922, *S. typhi* (clinical isolate) and *V. parahaemolyticus* XN89) exhibited inhibition zone from 2 to 3 mm (Table 2). Poor inhibition results (0–2 mm) were observed in other extract solvents, except for isopropanol extract that showed 4.0 and 3.5 mm inhibition zone for *S. aureus* ATCC25923 and *E. faecalis* ATCC2921, respectively, whereas the control of DMSO (10 *µ*l) showed no inhibition with any testing microorganism (Table 2).

### 3.4. Methanol Extract Minimum Inhibitory Concentration (MIC)

**Figure 3:**
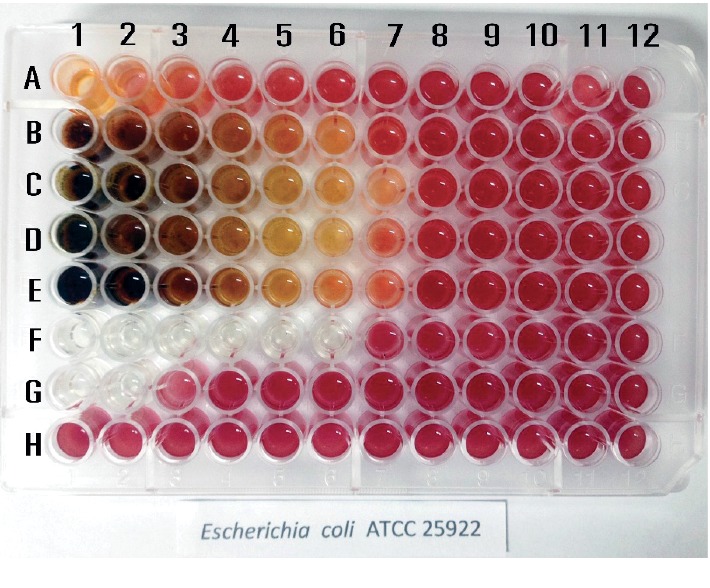
Minimum inhibitory concentration (MIC) sensitivity testing was a microdilution assay using flat shape 96-well plate. This is an example of crude metabolite extract *P. retrofractum* Vahl against *E. coli* ATCC25922. The MIC was read after tetrazolium was added which has shown the lowest concentration of antimicrobial agent at which it was colorless or changed to pale pink. The wells F1 to F6 were negative control, and the wells F7 to F12 were positive control.

Since the methanol extract showed the best inhibition result by disk diffusion test, minimum inhibitory concentration (MIC) was then performed for only the methanol extract. Details of MIC testing result are shown in Table 3. Overall, the results of the two methods were consistent. Lowest MIC values were 0.5 and 5.5 and 11 *µ*g/*µ*L in *C. albicans* ATCC90020, *B. subtilis* ATCC6633, and *S. typhi* (clinical isolate), respectively. In contrast, highest MIC values were 179.5 and 90 *µ*g/*µ*L in *P. aeruginosa* ATCC741 and *K. pneumonia* TISTR1843, respectively. MIC ranged from 22.5 to 45 *µ*g/*µ*L in the remaining isolates (Table 3). The colorimetric for bacterial detection technique was incubated at 37°C for 24 hr after inoculation, and infectious yeast *C. albican*s was at 37°C for 24 hr incubation. Then, 30 *µ*L tetrazolium salt (0.02 *µ*g/mL) was added into each of well. The colorimetric interpretation was displayed after the tetrazolium reaction with or without microorganisms within 1 hr after incubation. The color displayer was colorless with the microbial inhibition growth as a negative control in the wells F1 to F6 whereas microbial growth changed to a red color as a positive control in the well F7 to F12. The MIC was observed and read as the lowest concentration of antimicrobial agent at which no color changed or a bit color changing occurred starting from the well number 1 to the well number 12, respectively. The concentration of the row A to the row E (except row F1–6 was the negative control and F7–12 positive control) was using the two-fold dilution of various bioactive metabolite starting from the well number 1 to the well number 12, respectively. The row G tested for ampicillin and row H tested for DMSO (Figure 3). All of the wells from H1 to H 12 were of red colors; meanwhile, the amount of DMSO used for dissolubility of testing metabolite had no effect on microbial inhibition (Figure 3).

## 4. Discussion

Health problems have recently led to the development of natural antimicrobials to control microbial diseases. Medicinal plants and spices are one of the most commonly used natural antimicrobial agents in foods and have been used traditionally for thousands of years by many cultures for controlling common health complications. The WHO reported (2017, 2018) a list of bacteria for which new antibiotics are urgently needed for antibiotic-resistant “priority pathogens” of 12 families of bacteria that pose the greatest threat to human health. There were three families of critical priority 1 ranking: *Acinetobacter baumannii*, *Pseudomonas aeruginosa*, and *Enterobacteriaceae* include many harmless symbionts, and many of the more familiar pathogens, such as *Salmonella*, *Escherichia coli*, *Klebsiella*, and *Shigella,* six families of high risk priority 2 ranking which are *Enterococcus faecium*, *Staphylococcus aureus* (methicillin-resistant, vancomycin-intermediate and resistant, MRSA), *Helicobacter pylori*, *Campylobacter* spp., *Salmonellae* and *Neisseria gonorrhoeae*, and three families of medium risk priority 3 ranking which are *Streptococcus pneumoniae*, *Haemophilus influenza,* and *Shigella* spp., respectively [16].

Natural plant product-based antimicrobials drug discovery attained paramount importance as newly discovered drugs are likely to be effective against multidrug-resistant microbes. Bacteria such as *B. subtilis*, *S. aureus*, *E. faecalis*, *E. coli*, *K. pneumoniae*, *P. aeruginosa*, and *S. typhi* are multidrug resistant. Huge amount of antibiotics used for treating the diseases in human and animals result in this resistance. Multidrug-resistant bacteria are generated by accumulating the multiple genes encoding resistance to a single antibiotic in one cell. Besides that, overexpression of multidrug efflux pumps genes in bacteria could generate more resistant bacteria [17]. There have been many serious problems for treatment of these diseases in Thailand. Some of these microorganisms showed severe threatening for aquatic culture, too. For example, *V. parahaemolyticus* is a pathogenic microorganism in aquatic animals and is associated with contaminated food problem and causes food-borne disease. *V. parahaemolyticus* is an aquatic zoonotic agent. Humans are infected by consuming the contaminated seafood specially shrimp. This pathogen is orally transmitted in humans and causes gastroenteritis. In shrimp culture this pathogen causes acute hepatopancreatic necrosis disease (AHPND) and a very high mortality rate and economic losses [6]. Several toxins were reported to be produced by *V. parahaemolyticus* such as thermostable direct hemolysin (TDH), TDH-related hemolysin (TRH), and thermolabile hemolysin (TLH) [6, 7, 18]. The treatment of this severe human and aquatic pathogen is done by using the antibiotic. However, there were isolates from aquaculture which were resistant to antibiotics. Therefore, finding the alternative way for controlling this pathogen is necessary for human food and safety.

*C. albicans* was found as an infection on the mucous membranes of the intestinal tract and skin. This study showed that crude extract from the fruit of *P. retrofractum* Vahl using acetonitrile or methanol has a better potential to inhibit bacterial pathogens and *C. albicans.* The tested organisms, particularly Gram-negative organisms, showed high resistance towards different antibiotics generally. However, this study showed that they were inhibited by crude *P. retrofractum* Vahl extract even at a lower concentration.

Extraction of the bioactive compounds was reported by many researchers. These bioactive compounds belong to various groups such as tannins, alkaloids, glycosides, lignans, and terpinoids. The bioactive compounds are usually extracted by different solvents. The chemical properties and characterizations of these solvents could play a vital role in crude extraction property. Some factors should be considered when choosing the appropriate solvent in the study including solvent power (selectivity), boiling temperature, latent heat of vaporization, reactivity, viscosity, stability to heat, oxygen and light, and the last but not the least safety. In this study methanol showed better results as a solvent used in extraction. This might be related to the polarity index of methanol (5.1) [19]. Mostly, methanol is used to extract various polar compounds; however, certain non-polar groups are fairly soluble in this solvent. Furthermore, methanol has the lowest boiling point among all the alcohols. Therefore, the extraction and concentration of bioactive compounds can be more easily done by this solvent. In this research, the results showed that the amount of *P. retrofractum* Vahl crude extract by methanol was less than other solvents, and it might be illustrated that the concentration of active biocompounds in methanol extract might be more than others. In other words, the more bioactive compounds with higher concentrations were extracted by methanol. Further experiments to identify the components quantity of each solvent can help us for better understanding of *P. retrofractum* Vahl bioactive compounds against microbial pathogens.

In conclusion, *P. retrofractum* Vahl bioactive compounds extracted by methanol solvent have a better potential against pathogenic microorganisms. Our study showed that this crude extraction can be used as prebiotics in aquatic culture. Further study for identifying the pure bioactive compounds can lead to finding new antimicrobial agents for human and even aquatic culture. This research could open a new horizon and show promising results towards finding a new natural antibiotic for multidrug resistant-pathogens control with using endogenic Thai food.

## Figures and Tables

**Table 1:** Percent yield of crude extract from *P. retrofractum* by several solvent extraction.

Extraction	Crude extract obtained from fresh fruits of *Piper retrofractum* Vahl (250 g)				
	Met^*∗*^	Hex^*∗*^	Dic^*∗*^	Iso^*∗*^	Ace^*∗*^
Crude extract (g)	13.6812	7.2825	27.8815	30.9531	20.9458
% Yield	5.47	2.91	11.15	12.38	8.38

^*∗*^Met = methanol, Hex = hexane, Dic = dichloromethane, Iso = isopropanol, and Ace = acetonitrile.

**Table 2:** Inhibition zone of crude extract from *P. retrofractum* by agar disk diffusion assay.

Microorganism	Inhibition zone of crude extract from organic solvent (mm)						
	Hex^*∗*^ 51.84 *µ*g	Iso^*∗*^ 45.68 *µ*g	Dic^*∗*^ 51.20 *µ*g	Ace^*∗*^ 45.84 *µ*g	Met^*∗*^ 57.44 *µ*g	Amp^*∗*^ 2.00 *µ*g	DM^*∗*^ 10 *µ*l
*E. coli*	0.50	0.50	0.50	0.50	2.00	5.00	ni
*B. subtilis*	ni	ni	ni	1.00	0.50	13.00	ni
*E. faecalis*	ni	3.50	1.50	1.25	3.50	48.00	ni
*K. pneumonia*	ni	ni	ni	1.00	ni	7.00	ni
*P. aeruginosa*	ni	ni	ni	ni	4.00	18.00	ni
*S. typhi*	ni	0.50	0.00	0.50	2.00	44.00	ni
*S. aureus*	1.00	4.00	2.00	ni	6.00	54.00	ni
*V. parahaemolyticus* XN89	0.50	ni	1.00	1.00	3.00	34.00	ni
*V. paraparahaemolyticus* 5HP	0.00	ni	ni	1.00	0.50	37.00	ni
*C. albicans*	1.25	1.00	ni	1.00	8.00	53.00	ni

^*∗*^Hex = hexane, Iso = isopropanol, Dic = dichloromethane, Ace = acetonitrile, Met = methanol, Amp = ampicillin, and DM = DMSO; ni = no inhibition.

**Table 3:** Minimum inhibitory concentration (MIC) of crude extract by microdilution assay.

Antimicroorganism	Weight of crude extract from organic solvent (mg/ml)				
	Hex^*∗*^	Iso^*∗*^	Dic^*∗*^	Ace^*∗*^	Met^*∗*^
*E. coli*	Ni	18.00	80.00	36.00	22.50
*B. subtilis*	Ni	9.00	80.00	71.50	5.50
*E. faecalis*	ni	71.50	40.00	4.50	45.00
*K. pneumoniae*	ni	Ni	ni	143.50	90.00
*P. aeruginosa*	ni	Ni	ni	143.50	179.50
*S. typhi*	ni	9.00	10.00	9.00	11.00
*S. aureus*	ni	35.50	80.00	71.50	45.00
*V. parahaemolyticus* XN89	ni	71.50	80.00	71.50	45.00
*V. paraparahaemolyticus* 5HP	ni	18.00	40.00	71.50	22.50
*C. albicans*	2.50	10.00	15.00	1.00	0.50

^*∗*^Hex = hexane, Iso = isopropanol, Dic = dichloromethane, Ace = acetonitrile, Met = methanol, and ni = no inhibition.

## Data Availability

The data used to support the findings of this study are available from the corresponding author upon request.
